# The Treatment of Insanity, Ancient and Modern

**Published:** 1850-04-01

**Authors:** 


### Art. II.—THE TREATMENT OF INSANITY, ANCIENT AND MODERN.

The early history of medicine clearly proves that diseases which are now of ordinary occurrence were known to the ancients, and that they were well acquainted with many remedies for the treatment of them which we still esteem valuable. The reason of this is obvious. The practice of medicine was originally founded on observation and experience. We accordingly find that the priest-physicians—named the Asclepiadæ, on account of the Temples of Health in which they officiated—not only prescribed remedial measures to those who, for the cure of their diseases, resorted to the shrine of Æsculapius, but "noted down with great care," says the learned Dr. Adams, in his preliminary discourse to the Works of Hippocrates, published by the Sydenham Society, "the symptoms and issue of every case, from which observations they became great adepts in the art of prognosis.* Unshackled by authorities, the Greek and Arabian physicians looked to nature, and, as Dr. Bostock remarks, were necessarily diligent collectors of facts.† They thus unconsciously anticipated the spirit of that inductive system of philosophy, the principles of which were, many centuries afterwards, so clearly propounded by Lord Bacon. It would, therefore, be wrong to infer that before the age of Bacon philosophers were not guided by any idea of eliminating principles from an induction of facts. Hence, Dr. Adams observes—"Whatever may now be thought of his general views or pathology, all must admit that the mode of prosecuting the cultivation of medicine adopted by Hippocrates was in the true spirit of the inductive philosophy,—all his descriptions of disease are evidently derived from patient observation of its phenomena, and all his rules of practice are clearly based on experience."‡ The Arabian physicians, Rhases, Avicenna, Averrhoës, from the fragments preserved to us by Galen,§ and the recently collected writings of Paulus Ægineta,|| were, in their treatment of disease, clearly governed by the same inductive principles. Nay, without wishing to disturb the shade of Sir William Temple, by instituting a comparison between the knowledge of the Ancients and the Moderns, in as far as the science of medicine, and thereby provoking the genius of a Swift to retort upon us another "Battle of the Books," we will venture to affirm, that notwithstanding so much is said triumphantly upon the Baconian method of induction, medical authorities of the present age constantly disregard that guiding star which should direct them through the labyrinth of their investigations; and, consequently, it has been said, that in the present state of medical science we have "more false facts than false theories,"—a severe and, we are afraid, very just censure. To collect facts that may be relied

* The Genuine Works of Hippocrates, by Francis Adams, LL.D. 2 vols. London. Vol. i. p. 6.

† Bostock's Sketch of the History of Medicine. London, 1835. P. 120.

‡ Op. cit., p. 17.

§ Galen. Opuscula Varia Græce et Latinè. 4to, London, 1640.

|| The Seven Books of Paulus Ægineta. Translated by Francis Adams, LL.D. 3 vols. London, Sydenham Society, 1844.

upon, and to associate them consecutively, requires patience, diligence, and much critical acumen; but to hazard an ingenious theory requires little intellectual effort: the inventive faculty is always busy in the minds of ignorant people. Hence, it was profoundly observed by Mr. Mills, that speculations upon abstract causes—the nature of the mind and soul—by no means should be regarded as any indication of intellectual advancement, but rather the contrary; for "such inquiries stand at the very threshold of human knowledge." * Accordingly, we may lay it down as a principle, in the practice of medicine, that the more obscure may be the pathology of a disease, the more careful we should be in observing its phenomena, and collecting such data as may guide us in its treatment; in order to illustrate which we may carry our induction back to such facts as were observed in the remotest ages, associating them with the modes of treatment which are at present conceived to be the most enlightened.

Among the diseases above referred to as having been known to the ancients, insanity, in almost all its phases, appears to have been well marked; and the retrospect is interesting and useful, as evincing the early recognition of the principles upon which the disease is still treated. We have many examples of mental disease recorded in the Scriptures. Saul was afflicted with melancholy,† and after taking what remedies‡ his servants and physicians prescribed, finding his case beyond remedy, it was conceived to be a visitation from on high.§ When Saul had been soothed, and refreshed, and calmed by music from the harp of David, the "evil Spirit"—which undoubtedly means the paroxysm—passed away; and the effect of music in such cases is still well known and taken advantage of in many public and private asylums. The disease was so far common, that David feigned himself mad, when, to escape the wrath of Saul, he sought the protection of Achish, the king of Gath.|| "Have I need of madmen," said Achish, "that ye have brought this fellow to play the madman to me?" Again, the learned Calmet recognises, in the imaginary transformation of Nebuchadnezzar, that form of monomania designated Lycantrophy, which, he observes, accounts satisfactorily for all that Scripture has recorded of him.¶ Upon the

* Mills's (John Stuart) System of Logic, Ratiocinative and Inductive. 2 vols, 8vo. London, 1846. Vol. i. p. 200.

† 1 Samuel, xvi. 23. ‡ Genesis, l. 2.

§ Calmet's Dictionary, by Taylor. 5 vols. 4to. 1841. Art., "Saul."

|| 1 Samuel, xxi. 10—15.

¶ Calmet, op. cit. Art., "Nebuchadnezzar."

same principle, Dr. Mead explains the possession of the demoniac described in St. Luke.* A very particular account of this disease was given by Marcellus di Sida, a Greek physician, fragments of whose writings have been preserved by Ætius and Oribasius. He states that the malady is commonly aggravated at the approach of spring, as in the month of February; and he adds, that in certain countries it is sometimes epidemic.† Those labouring under Lycantropia, observes Paulus Ægineta, "go out during the night imitating wolves in all things, and lingering about sepulchres until morning. You may recognise such persons by these marks—they are pale, their vision feeble, their eyes dry, tongue very dry, and the flow of the saliva is stopped; but they are thirsty, and their legs have incurable ulcerations from frequent falls. Such are the marks of the disease. You must know that lycantrophy is a species of melancholy, which you may cure about the time of the attack, by opening a vein and abstracting blood to fainting, and giving the patient a diet of wholesome food. Let him use baths of sweet water and then milk-whey for three days, and purging with the hiera from the colocynth twice or thrice."‡ The absurd imaginations of persons, says Dr. Arnold, afflicted with this species of insanity are almost innumerable; and he cites authorities for persons who have believed themselves transformed into wolves, dogs, lions, cats, cows, oxen, &c.§ We recently read in a foreign journal, an account of some young women, secluded in a convent, who fancied themselves metamorphosed into cats, and on a sudden, when at their devotions, unanimously set up a hideous mewing and screaming. However strange or incredible the extraordinary fancies of such monomaniacs may appear, we must take into consideration the influence of moral causes on the insane mind. "Two centuries ago, persons were everywhere to be found," observes Dr. Pritchard, "who fancied themselves to be possessed by demons, just as the ancients were pursued and agitated by the furies. Dæmonomania occupies, therefore (as well as lycantrophy), an important place among the forms of insanity described by old writers; and we are informed by Jacobi, that this is still the character which, in some catholic countries, insanity connected with superstition frequently assumes. 'In modern times,' says M. Esquirol, 'the punishments which the priest denounces have ceased to influence the minds and conduct of men, and govern-

* Mead, Opera Medica. 2 tom. Gott. 1748. Vol. i.

† Sprengel, Hist. de la Médicine. 9 tom. Paris, 1815. Vol. ii. pp. 174, 175.

‡ Paulus Ægeneta, op. cit. vol. i. p. 389.

§ Arnold's Observations on Insanity. 2 vols. London, 1806. Vol. i. p. 122.

ments have recourse to restraints of a different kind. Many lunatics express as much dread now of tribunals of justice as they formerly entertained of the influence of stars and demons.' Such unfortunate persons are always acted upon by apprehension, fear, and terror, as if such were the veritable causes of their preternatural possession. Thus, an individual is now at the Petites Maisons because he is afraid of the police, who would formerly have burned him for being a devil."* There is nothing so very extraordinary in the origin of the notion of demoniacal possession, when we remember that there was formerly a more or less direct intercourse supposed to exist between the material and spiritual world. Hence Dr. Davis, in his Introduction to "Pinel's Treatise on Insanity," truly remarks,—

"The mythologists, who maintained the existence of spirits of different orders and qualities, believed that, in some instances, the intellectual faculty was merely deranged by the malignant influence of a demon; and in others, where the change of character was more evident and complete, they imagined an actual exchange of the indwelling soul to have taken place, and the maniac was consulted as the organ of an oracular spirit, or shunned as embodying an emissary of the evil principle. Accordingly, in all countries remarkable for their superstition, the treatment of mental affections has been associated with other duties of the sacerdotal office. At both extremities of ancient Egypt—a country at that time exceedingly populous and flourishing—temples were dedicated to Saturn, whither melancholics resorted in quest of relief. Whatever gifts of nature or productions of art were calculated to impress the imagination, were there united to the solemnities of a splendid and imposing superstition."†

Nor is it at all surprising that such erroneous notions should have been entertained in those early ages respecting the nature of insanity, when we find, even in our own time, men of scientific eminence prejudiced by feelings which would appear to be of the same description. It is very remarkable that Dr. Burrows, who had so much experience a few years ago in this department of the profession, should, in the Introduction to his Commentaries—which is, nevertheless, an excellent practical work,—thus express himself respecting the nature of insanity: "Madness is one of the *curses* imposed by the wrath of the Almighty on his people for their sins; and deliverance from it is not the least of the miracles performed by

* Pritchard's Treatise on Insanity. London, 1835. P. 31.

† Dr. Davis, Introduction to Pinel's Treatise on Insanity. Sheffield, 1806. P. xxiii.

our Saviour."* We are certainly taught to believe that the fall of our first parents brought "death into the world, with all our woe," but why madness should be singled out as the instrument of Divine wrath, any more than any other disease, we are at a loss to conceive: besides, the very foundation of Christianity itself, being a great social revolution in the progress of civilization, so far from being a deliverance from the calamity, rather augmented its diffusion; and we therefore find the accomplished and eloquent historian, Dr. Robertson, truly observe:—

"When the human mind is roused by grand objects and agitated by strong passions, its operations acquire such force, that they are apt to become irregular and extravagant. Upon any great revolution in religion, such irregularities abound most at that particular period when, men having thrown off the yoke of their ancient principles, do not yet fully comprehend the nature, or feel the obligation, of those new tenets which they have embraced. The mind in that situation, pushing forward with the boldness which prompted it to reject established opinions, and not guided by a clear knowledge of the system substituted in their place, disdains all restraint, and runs into wild notions, which often yield to scandalous and immoral conduct. Such was the effect in the first ages of Christianity, as well as at the era of the Reformation. The renunciation of the ancient faith, and ignorance of that which they had embraced, excited converts to acts more resembling insanity than that of that religion which inculcates the purest morality and government of our passions."†

The truth, however, would appear to be this: that the phenomena of insanity, even in the contemplation of an educated mind, are so perplexing and startling, and the aspect of a person in a state of mania is frequently so dreadful, that humanity recoils from so sad a spectacle, and becomes unconsciously agitated by superstitious emotions. Not many months ago it was our painful duty to attend, on the Continent, a lady to whom we were called in consultation, she having been for several days suffering under an acute attack of mania. One of the most eminent physicians in Prussian Germany was in consultation with us; and we were together examining the patient, when suddenly she started and made a dash at the window, through which she would inevitably have precipitated herself into the street, had we not, single handed,

* Dr. Burrows on the Causes, Forms, Treatment, &c., of Insanity. London, 1828. Introduction, p. i.

† Robertson's History of Charles V. Dugald Stewart's edition, 12 vols. London, 1817. Vol. iv. p. 60.

and not without difficulty, held her back. The attendants, deceived by her apparent tranquillity, had left us in consultation; and the instant our *confrère* found himself called upon to help us, by holding the patient, he retreated; and afterwards assured us that he could not, under any circumstances, reconcile himself to touch an insane person. He could not explain his dread; but allowed that it was a feeling that existed instinctively in his nature, which he could not control.

The ancients describe melancholia and lycantropia more fully than mania; the phrensy, described by Hippocrates—phrenitis—was febrile delirium;* but in some cases, obviously chronic mania;—thus, in one of his Aphorisms, he states—" Persons above forty years of age who are affected with phrensy, do not readily recover."† The greater number, however, of physicians among the ancients conceived madness and melancholy to be only modifications of the same disease, differing "*intenso et remisso gradu.*" Burton, in his " Anatomy of Melancholy," has, with his usual research, collected a host of curious facts and anecdotes illustrating the different forms of this disease, under which he was himself, says Mr. Granger, a sufferer, "for he composed this book with a view to relieve his own melancholy; but increased it to such a degree, that nothing could make him laugh but going to the bridge-foot, and hearing the ribaldry of the bargemen, which rarely failed to throw him into a violent fit of laughter. Before he was overcome with this horrid disorder, he, in the intervals of his vapours, was esteemed one of the most facetious companions in the university."‡ We learn from Galen, that mania was supposed to be connected with inflammation of the brain; and melancholia, with derangement of the pyloric and cardiac extremities of the stomach. Their treatment may be said to have been, as in the present day, medical, physical, and moral. They also used coercion. " Above all things," says Paulus Ægineta, " persons affected with mania must be secured in bed, so that they may not be able to injure themselves, or those who approach them; or swing in a wicker-basket, or a small couch, suspended from on high."§

The treatment which Celsus describes as proper in cases of insanity merits particular attention; it is in every respect interesting, involving many principles of practice which are still recognised. " It is unnecessary," says Celsus, " to oppress those with very harsh

* Hippocrates, op. cit. pp. 356, 357. † Aphorism 82.

‡ The Anatomy of Melancholy. 2 vols. London, 1837. Vol. i., p. 134.

§ Paulus Ægineta, op. cit. vol. i. p. 385.

coercive measures whose malady only extends to words, or even trifling assaults with the hands; but it is proper to confine those who conduct themselves violently, lest they may injure themselves or any other person."

"The ancients," he tells us, "recommended such patients to be kept in darkness, and judged that darkness itself tended to tranquillize the mind. But Asclepiades thought darkness might excite so much terror, that he advised them to be kept in the light. Neither should be without exception. It is best to try each, and to keep him in the light who dreads darkness, and him in darkness who dreads the light. It is absurd to apply remedies where the furor is at its height; but when the case admits of relief, no time should be lost. Asclepiades has characterised blood-letting as tantamount to murder; but if the patient's strength permit, he ought to be bled. It requires deliberation about the administration of injections. After the interposition of a day, it will be proper to shave the head, and then foment it with warm water in which vervains have been boiled,—with astringent remedies; or to foment it first, then to remove the hair and again foment; and lastly, to embrocate the head and nostrils with rose-oil. * * * Such is the treatment to be pursued towards those who are not debilitated; but if there be weakness, the head is only to be moistened with rose-oil, to which some wild thyme or something similar has been added."

"It is necessary," continues Celsus, "to conduct ourselves towards all insane persons in a manner that shall be suitable to the nature, disposition, and habits of each. The groundless apprehensions of some must be alleviated; as it happened to a very rich man who dreaded starvation, and who changed his purpose when made to believe that he had acquired hypothetical possessions. The audacity of others requires coercion, as is done with those persons in restraining whom even stripes are applied. Yet we should assent to them more frequently than oppose them, by which means the mind will be gradually brought from an irrational to a more rational method of discourse. Sometimes the mental energies of the patient should be elicited; as is done with literary men, to whom a book is read, either with a propriety of accentuation, if they be pleased with it, or in a perverted manner, if that itself offend them; for by their emendations they begin to reason. Some have been brought to eat, who had previously refused, by being placed among those who were feasting. To all persons so affected it is very difficult to obtain sleep, which is above all things necessary; for after this, many begin to recover. Saffron oil, with iris, on the head, aids in producing sleep, and also in tranquillizing the mind; but if they continue vigilant, some procure sleep by giving them a decoction of poppies or henbane to drink. * * * Nor is it improper, if blood have not been previously let, to apply cupping-glasses to the incised occiput when there is continued vigilance and delirium, which will relieve the disease and procure sleep. There is another species of insanity which admits of a longer duration, because, for the most part, it begins without fever. Blood-letting in this is beneficial; if any circumstance impede this, the first remedy is abstinence, the second is to purge with white hellebore, and the third is to administer a vomit.*

The medical treatment here described by Celsus, corresponds in a very remarkable manner with the treatment which is still found to be most effective in this disease. The moral management, too, is recommended to be mild and persuasive, excepting in case of furor, simulating that described by Sophocles, where Ajax acts the part of a madman among the shepherds and cattle of his rival Ulysses; and by Euripides where Orestes is affected with madness after the murder of his mother. In such cases, correction and coercion are prescribed. "When he has said or done anything wrong, he must," observes Celsus, "be chastised by hunger, chains, and stripes. He must be made to attend, and to learn off something that he may remember, for by this it will happen that by degrees he will be led to consider what he may be doing. It is also beneficial, in this malady, to be put into sudden dread, for a change may be effected by withdrawing the mind from that state in which it had already been."† We are not, however, to infer that these extreme measures were resorted to in any other cases than those of extreme furor. We find, indeed, that Cœlius Aurelianus, a physician, a few years before Galen, complained loudly of unnecessary restraints and cruelties being inflicted upon lunatics. He was the Pinel of his age.‡

From the early history of medicine, we learn, as we premised, that the ancients were in possession of many valuable remedies which they used in the treatment of insanity. The most ancient Greek physician with whom we are acquainted, Melampus of Argos, cured the daughter of King Prœtus, of melancholia, by bathing and the administration of hellebore.§ Venesection was also a remedy of very ancient origin, for Podalirius, on his return from the Trojan war,

* Cornelius Celseus de Medicina. Lib. iii. cap. xviii.

‡ Galen, op. cit.

§ Although Ovid in his Metamorphosis has recorded this cure, we imagine it not altogether a myth. See Strabo, lib. ix.; Herodotus, book ix. c. 33; Pliny, Hist. Nat., lib. x. c. 49.

cured the daughter of Damethus, who had fallen from a height, by bleeding her in both arms; and the practice of incision and scarification was employed in the Grecian camps before Troy. The followers of Hippocrates opened veins in the arms, feet, temples, tongue, &c., and cupped in almost every part of the body.* Opium, or some preparation of the poppy, was certainly known in the earliest ages; it has been conjectured that it was opium which Helen mixed with wine and gave to the guests of Menelaus, under the expressive name of Nepenthe, to drive away their cares, and the conjecture certainly receives some support from the fact that the Nepenthe of Homer was obtained from the Egyptian Thebes—hence the tincture of opium was called the Thebaic tincture.†

Hyoscyamus was used by Hippocrates; and Celsus, as we have seen, prescribed a decoction either of henbane or of poppies to produce sleep in mania.‡ The most favourite, and apparently successful remedy employed in the treatment of mania and melancholy was hellebore. The Greek, Arabian, and Roman physicians held it to be an unfailing specific; and so celebrated were its effects, that many learned modern authors have come to the conclusion, that we have lost sight of the remedy which went by that name. This would appear to be the opinion of Dr. Arnold, and Dr. Lorry, who, referring to this method of cure under the name of *helleborism*, laments "the unhappy lot of the modern insane," who have no longer the advantage of a "certain cure."§ The classical reader will remember many allusions to hellebore, and the far-famed Anticyris islands where it was gathered. Horace, after giving a lively description of a madman who fancied himself attending a show, followed by a troop of comedians, and impersonating actor and spectator, adds,—

"He when his friends, at much expense and pains,
Had amply purged with hellebore his brains,
Come to himself. 'Ah, cruel friends!' he cried;
'Is this to save me? Better far have died,
Than thus be robbed of pleasure so refined,
The dear delusion of a raptured mind.'"||

So also Perseus tells Nero, in the sixteenth verse of his fourth Satire, that instead of taking upon himself the heavy responsibility

* Le Clerc. Hist. de la Medecine. 4to. Amsterdam, 1702. Tome i.

† Paris Pharmacologia. London, 1843. P. 11.

‡ "Quidam somnum molliunter potui dando aquam in qua papaver aut hyoscyamus decocta sit."—*Loc. cit.*

§ Observations, p. 4, et seq.

|| Francis Horace.

of government, which demanded more experience and sound judgment than he possessed, it were better for him to take powerful medicines to clear his understanding :—

> "Thou hast not strength such labours to sustain :
> Drink hellebore, my boy,—drink deep ! and purge thy brain."*

Dr. Cox, in his "Practical Observations on Insanity," accuses Dr. Lorry of having "betrayed an undue veneration for the ancients at the expense of the moderns," in giving credence to the infallibility of this remedy ; and suggests that, "had the ancients been in possession of calomel and tartarized antimony, it is probable that they would have preferred them to hellebore; for," he adds, "this celebrated root seems to have possessed no specific virtue—no antimaniacal property; but to have acted on the stomach and bowels."† This is somewhat of an assumption.

The hellebore used by the ancients in maniacal cases is now proved to be identical with our *veratrum album ;* and the anomalous effects produced by this and other electro-stimulants on the nervous system are well-known. It is obvious that it did more than act on the stomach and bowels ; for Hippocrates expressly says, in his sixteenth aphorism, "Hellebore is dangerous to persons whose flesh is sound, for it induces convulsions."‡ He prescribed it in tetanus, and gives a variety of cautions respecting its administration ; in fact, recent experience proves that veratria is a valuable remedy in many nervous affections. The action of sabadilla, another electro-stimulant, is equally remarkable. Plenck reports the case of a young man who was rendered insane by the application of sabadilla powder to the temples.§

The ancients, on account of their ignorance of pathology and therapeutics, used these different remedies, to a certain extent, empirically; but guided by observation and experience, they were, probably, as successful in the treatment of insanity as in the treatment of other diseases. Their mistaken notions respecting the functions of the brain and heart, and the properties of medicines generally, to which they attributed certain occult qualities, did not mislead or blind their judgment as to the positive effects arising from their administration. The progress of medical science places the modern physician in a very different position; he can reason therapeutically

* Dryden.

† Practical Observations on Insanity, by Joseph Mason Cox, M.D. London, 1806. Note, page 7.

‡ Hippocrates' Works, op. cit., p. 725. § Murray, Materia Medica. *App.*

on the *modus operandi* of the medicines he prescribes, and in all cases adapt these to the morbid action, or condition of the organ that may be affected. Without being guided by this twofold principle, he would be prescribing in the dark, and would resemble Swift's Apothecary—" pouring bodies of which he knows little into a body of which he knows less." The modern treatment of insanity must depend, therefore, on the pathological view we take of the cause of the disease, which explains the very contrary and antagonistic modes of treatment which have been pursued by different physicians of eminence. Does the disease arise from an excessive quantity of blood in the brain ? or from some irregularity or disturbance in its local distribution ? If so, venesection, to a certain extent, may be clearly indicated. Does it depend on some derangement of the digestive functions, acting by sympathy on the brain ? If so, cathartics and alteratives may effect a cure; and such cases may be cited in support of the views of those French pathologists who consider the abdominal viscera the seat of the disease. Again; does it arise from debility—from loss of blood ? Hæmorrhage has been known to produce insanity ? In that case, tonics and stimulants are necessary. Hufeland relates the case of a boy between thirteen and fourteen years of age who suddenly began to talk in a wild and incoherent manner, and became ungovernable. This state was assuaged by soporifics; but the paroxysm was observed to recur whenever he was placed on his feet. When examined, a reddish spot was noticed on one foot, which, when pressed, occasioned a fresh paroxysm, and, upon an incision being made, a minute piece of glass was discovered and extracted. The boy was furious during the operation, but every symptom of violence vanished when the offending cause was removed.* " Insanity," observes Dr. Battie very truly, " discovers so much variety with respect to its causes and circumstances, that, like any other disease, it rejects all general methods of cure."† " A variety of medicines," says Dr. Charlesworth, physician to the Lincoln Lunatic Asylum, " has been employed with a view to the cure or abatement of insanity, without peculiar results. The causes of insanity are so various, and it arises from so many different local affections of the head, stomach, and alimentary canal, requiring different modes of treatment, that the recommendation of any single medicine is impossible."‡ Nevertheless, physicians have not failed to

* Hufeland *apud* Burrows, op. cit. p. 211.

† Treatise on Madness, by William Battie, M.D. London, 1758. P. 94.

‡ Remarks on the Treatment of the Insane, by E. P. Charlesworth, M.D. London, 1828. P. 3.

attach special importance to certain medicines, all of which are doubtless valuable, but not one can be considered specific, nor upon any *one* ought any practitioner to pin an exclusive faith. The following table, which we might extend to an indefinite length, exhibits the authorities in favour of the following narcotics and stimulants :—

Opium is recommended by Odier, Cox, Brandreth, Chiarruggi, Dæmling, Reil.

Hyoscyamus, by Fothergill, Willis, Stoërck, Selig, Meyer, Hufeland.

Belladonna, by Theussenck, Vogel, Buchoz, Ludwig, Remer, Schmalz, Hufeland, Franck.

Stramonium, by Allioni, Maret, Stoërck, Remer, Reel, Greding, Schneider, Hufeland, Burrows.

Digitalis, by Dariom, Ferriar, Currie, Fonzago, Jones, Muller, Guislain, Morus, Burrows, Ellis, Hallaran, Pritchard.

Tartar Emetic, by Willich, Muller, Bodel, Frize, Burserius, Baldinger, Hufeland, Fordyce.

Camphor, by Wherlhof, Lobenstein, Kinneir, Remer, Avenbrügger, Perfect, Percival, Hufeland, Copland.

Musk, by Thilericus, Locher, Selig, Pargetier, Gmelin.

We believe that each of these remedies, and many others which we could add, are valuable, but no general rule can be laid down for their administration, for no two cases of insanity are alike, and the physician must be guided by the peculiarities of the symptoms he may observe in each. Without, however, having a clear pathological notion of the state of the circulating and nervous systems, we repeat, it is impossible to prescribe upon philosophical principles.

As preliminary measures to ensure the cure of insanity, all physicians, ancient and modern, have agreed on the necessity of early treatment and removal from home. "*Recens curationen*," observes Celsus, "*non habet difficilem.*" So also Piso and Avicenna, "*Circa initia diligentissime curanda est melancholia.*" Dr. Willis, in his evidence before the Committee of the House of Commons, relating to the case of George III., stated that nine out of ten patients placed under his care within three months after they had begun to be mentally affected, recovered. Dr. Burrows reported 221 cures out of 242 recent cases. Dr. Finch stated that sixty-one out of sixty-nine patients recovered who were placed in his asylum within three months after the attack. From the reports of the Retreat near York, seven out of eight recent cases terminated in recovery. M. Pinel states, that the greatest number of recoveries takes place during the first month, and the average duration of the disease is six months. The statistical returns throughout Great Britain, France, Germany, and America, prove the same fact; therefore it behoves the relations and friends of persons so affected to watch the stage of incubation. We have already published a distinct memoir on this very important subject.

Removal from home is also imperatively necessary. "This," says Haslam, "should be enforced as early in the complaint as possible. During the continuance of the patient in his own house, he can never be kept tranquil. The interruption of his family; the loss of the accustomed obedience of his servants; the idea of being under restraints in a place where he considers himself the master, will be constant sources of irritation in his mind. It is also known, from considerable experience, that of those patients who have remained under the immediate care of their relations and friends, very few have recovered."* When Dr. Willis undertook the care of George III., he considered an alteration of surrounding circumstances so essential, that he changed, not only the furniture of the king's apartments, but also his attendants.

Esquirol assigns the following reasons for the seclusion of the insane: 1st, for their own safety, as well as for that of their family or the public; 2nd, for the purpose of withdrawing them from causes of excitement; 3rd, in order to overcome resistance to the means of cure; and 4th, for the purpose of subjecting them to the regimen adapted to their condition.† Numerous facts, observes Esquirol, prove that confinement alone has cured many insane persons, and that it has sometimes produced this effect instantaneously.‡ We had recently a patient under our own care who was always well conducted with us after the first few days of his return; but whenever he went home, the malady returned, from some local associations, probably, which affected the mind.

Here we may be asked whether modern physicians have made any progress whatever in the successful treatment of insanity? And this inquiry we should feel inclined to answer affirmatively, although no uniform system of practice has been, for very obvious reasons, agreed upon. We no longer hear, in any asylum, of periodical venesection and vomits—chains and stripes. Dr. F. Monro, in his evidence respecting the treatment of the patients in Bethlem, before the Committee of the House of Commons, in 1815, states that "patients are ordered to be bled about the latter end of May, or beginning of May,

* Haslam, Observations on Insanity. London, 1798. P. 133.

† Esquirol, Des Maladies Mentales. 2 tom. Paris, 1838. Tom. i. p. 40.

‡ Esquirol, Observations on the Illusions of the Insane. Translated by Liddell. London, 1833. P. 82.

*according to the weather.* After they have been bled, they take vomits once a week for a certain number of weeks; after that we purge them. *That has been the practice invariably, for years before my time; it was handed down to me by my father, and I do not know a better practice.*"* "The period of physicing," says Haslam, in the same Report, "continues from the middle of May, regulated by the season, to the latter end of September. Two bleedings, according to discretion; half-a-dozen emetics, if there should be no impediment to their exhibition; and the remainder of the time, till Michaelmas, a cathartic once a week."† "The curable patients," says Crowther, "are regularly bled about the commencement of June, or latter end of July; I have bled 150 patients at a time."‡ We find Dr. Willis advise, as the first indications in the curative process of madness,—manacles, fetters, and stripes; he also recommends "that the food should be slender, and not over-delicate, clothing rough, bed hard, and treatment severe and rigid. Those labouring under obstinate madness, were rarely submitted to any curative means."§ With this evidence before us, we may fairly consider that we have made some advancement in the medical and moral treatment of the insane,—at all events, we "know a better practice" than that which had been handed down to Dr. Monro by his forefathers.

The Commissioners in Lunacy, in their last Report, have published a full and satisfactory account of the medical treatment which is adopted in the lunatic asylums under their surveillance. They addressed an official circular to the proprietors or superintendents of them, requiring to be informed of their "methods of treating insanity and the disorders complicated with it,—1st. In mania; 2nd. In epilepsy connected with insanity; 3rd. In paralysis connected with insanity; 4th. In melancholia." They likewise requested medical officers to give them accurate information as to the result of their experience in the employment of particular remedies,—such as blood-letting, general or topical; emetics; purgatives; antimonials; opiates, or anodynes of any kind; antispasmodics; tonics; stimulants; and hot and cold bathing, respectively; and also to communicate their observations as to the nature of the diet and regimen which have been found by them most beneficial in the treatment of

* Reports, No. 1. Minutes of Evidence taken before the Select Committee appointed to consider of provision being made for the better regulation of mad houses in England, May, 1815, p. 95. † Ibid. p. 63.

‡ Practical Remarks on Insanity. By Bryan Crowther. London, 1811.

§ Vide Burrows' Commentaries, p. 687.

insanity in its various forms." To this circular, the Commissioners received answers from fifty-three asylums; so that we have before us a complete view of the medical treatment now adopted. It is, perhaps, to be regretted that these answers contain so few allusions to the pathological views entertained by the several writers. General bleeding is, upon the whole, much condemned. Dementia and incurable insanity are often the result of injudicious bleeding. Violent paroxysms of acute mania depend, according to Dr. Sutherland, not on cerebral inflammation, but on irritation; and the arterial congestion found in post-mortem examinations in such cases, he conceives not to be the result of inflammation. Here we at once come to the *questio vexata.* The experience of Foville, in one of the largest lunatic asylums in France, is in favour of general and topical bleeding; and the views of the late Dr. Pritchard entirely coincide with those of M. Foville. "My own experience," he observes, "has afforded me sufficient opportunities of forming opinions on the effects of remedies in insanity, since, for twenty years, I have never been without patients labouring under that disease, who were, more or less, under my care and observation. Long before Dr. Foville's remarks were known to me, I had pursued the practice which he has recommended, having been led to adopt it by similar considerations," [pathological appearances indicating inflammatory action;] "and I entertain no doubt of its practical advantages. I am very far from approving or wishing to recommend such detractions of blood as those which appear to have been practised by Dr. Rush; but I have been convinced, by the evidence of numerous facts, with respect to which I could not be mistaken, that bleeding, both local and general, is, under due limitations, serviceable in cases of insanity."* We have no doubt, from our own observation, that cases may occur in which bleeding may be serviceable, and in a strong plethoric habit cut short an attack of mania; but the after consequences are of course to be duly considered. "The quantity of blood," says Sir Alexander Morison, "must be regulated by circumstances; the plethoric and the cachectic; the strong and the weak; difference of sex; constitutional varieties; the highly and moderately excited, all demand consideration, and require different measures. One case may require the loss of sixteen or twenty, another only ten, eight, or six ounces of blood, or even a smaller quantity."† The advantage to be derived from topical

* Dr. Pritchard, Treatise on Insanity. London, 1835. P. 258.

† Sir Alexander Morison, M.D., Outlines of Lectures, edited by his son, Thomas Coutts Morison. London, 1848. P. 337.

bleeding appears to be, however, admitted generally, in the evidence before us. After all, we can but agree with the verdict which Dr. Copland pronounces on these conflicting opinions:—

"In estimating," he observes, "the opinions of physicians attached to public institutions for the insane, as to the propriety or extent of vascular depletions, the sphere of their practice should not be altogether unheeded, and especially the circumstances of the patients having been treated previous to admission, and the duration of those which have been called recent cases. It is very obvious, that a patient who has been ill only three or four days, but during that time has been very actively treated, will not bear evacuating means on admission into an asylum; whilst another case, that would have been benefited by vascular depletions in the first few weeks of the malady, may be injured by them after a week or a fortnight had elapsed. After all that can be advanced on this point, the propriety of prescribing sanguineous depletion, to whatever extent, must depend upon the pathological knowledge and discrimination of the physician; and if he possess not these qualifications in a high degree, and unless he study and practice his profession as a whole, and as a profound and comprehensive science, and not as a trade or mechanical art, divisible into a number of separate parts, he cannot truly possess them—he is quite incapable of rationally and judiciously treating insanity or any other class of maladies."*

Whatever views may be entertained concerning the proximate cause of insanity, there can be no doubt that the immediate indications of treatment are to equalize and modify the circulation, particularly within the brain, and tranquillize the nervous system. Whether the disease depend upon an excess in the quantity of blood in the brain, on a want of equilibrium between the arterial and venous circulation,† on its local distribution, on the rate or manner of circulation, or on any alteration in the constituents and qualities of the blood itself,‡ are inquiries of much interest and great difficulty. "The history of all cerebral disease, and the examination of those instances where it has been fatal," observes Dr. Holland, "show the remarkable influence of these several conditions; and particularly how small an amount of obvious change in the circulation, as in the slighter degrees of inflammation of the membranes, is capable of producing great disturbance in the mental functions."§ The effect of warm

* Copland's Dictionary of Practical Medicine. London, 1844. Vol. ii. Art. "Insanity."

† Abercrombie, Pathological and Practical Researches on Diseases of the Brain and Spinal Cord. Edinburgh, 1828.

‡ Burnett, "Insanity tested by Science." London, 1848.

§ Holland, Medical Notes and Reflections. London, 1840. P. 242.

and cold bathing, under such circumstances, is very obvious, and has in all ages been recognised. Celsus advises us to begin with hot, and then proceed by degrees to tepid water; and, lastly, to pour cold water over the head and whole body; then to dry and to anoint. He expressly says,—"It is very beneficial for one who has a weak head to hold it where a strong stream of water may fall on it:"* hence the douche is a remedy of great antiquity. Hoffman recommends warm bathing in the following terms:—"It is not from reason, but from a long course of experience, that we assert the excellence of this remedy in maniacal cases; for we have seen numerous instances of inveterate melancholy and raving madness happily cured by its means, after the use of bleeding, diluting medicines, and medicines consisting chiefly of nitre. And this kind of cure I have recommended to many foreign physicians, who, as well as myself, find it highly serviceable and beneficial. Whence I have often wondered that this method of cure for madness should be so much neglected in our time; whilst bathing has from the earliest ages been employed for this purpose, insomuch that the ancient physicians had recourse to it as a thing they entirely depended upon."† According to Poggius, the Florentine, the insane were in his time placed in warm baths up to the knees, waist, or armpits, with the view of drawing the blood downwards, whilst the head was left exposed. Pomme treated maniacs by employing the warm baths for eight hours every day, and applying at the same time cloths wet with cold water to the head. He even kept them in the bath for twenty-four hours.‡ Pinel introduced Pomme's plan in France, ordering that the patient should sit in a warm bath, while a column of cold water descended upon the head, varying in height according to the effect desired. Sometimes he limited this to a mere sprinkling (*douche en arrosoir*); the intention being to drive the circulation towards the surface, and diminish, by refrigeration, the energy of the brain. This, however, is a remedy which should be applied with great caution. Esquirol affirms that he has known disorganization of the cerebrum produced by the shock of the douche, and madness thereby rendered incurable. He tried it on his own person, and thus expresses himself:—"It appeared to me as if a column of ice were broken on the parts, but the pain was much more acute when the stream fell on the front parietal suture; it was more supportable on the occiput."§ Hence,

* Com. Celsus, op. cit.

† Hoffmann, New Experiments and Observations upon Mineral Waters, with Notes by Shaw. London, 1731. P. 188.

‡ Orteschi, Giornel Med., tom. ii. p. 100.

§ Esquirol, op. cit., tom. ii. c. 75.

when the douche is applied, patients are generally observed to present the back of the head to the stream. In the Commissioners' Report, the evidence is favourable to the employment of warm and cold bathing; but the shower is, upon the whole, recommended in preference to the douche, which we have ourselves known to produce violent reaction. Brierre de Boismont has recently revived the plan adopted by Pomme. The following is a *resumé* of the conclusions to which he has come, extracted from a Memoir just submitted to the French Academy:—

"1. Every acute form of insanity, mania particularly, may be cured within one or two weeks.

"2. The treatment employed to obtain this result, consists in prolonged baths and continued irrigations.

"3. The duration of the bath ought to be from ten to twelve hours, but may be prolonged to fifteen or eighteen hours.

"4. The irrigation accompanying these baths should be continued the whole time, but may be suspended when the patient becomes tranquil.

"5. When patients have had eight or ten baths without any relief or improvement in their appearance, they may be discontinued, but at a future period renewed.

"6. The bath should be given at a temperature of 28° or 30° centigrade, and the irrigations at 15°.

"7. Cases of recent acute mania yield most readily to this treatment; next come simple acute delirium—insanity from drunkenness—[*le délire des ivrognes*]—puerperal mania—melancholia; but these cases are not relieved so readily as acute mania.

"8. Chronic mania, with agitation and intermitting mania, have been improved, but not cured, by this treatment. It has not been tried in cases of mania combined with paralysis and epilepsy.

"9. Facts recently collected since the publication of this memoir, prove its success in cases of hysteria and certain nervous diseases, without mania.

"10. From the above facts, it may be affirmed that cases of acute mania are more readily cured by this than any other form of treatment.

"Lastly. The employment of prolonged baths is by no means new in science; but this plan of treatment, which is so easy, and which might be tried everywhere, has not been generally applied in such cases."*

The beneficial effects of sedatives in tranquillizing the nervous system and curing insanity, are recognised and attested by *every authority* in the report before us, and fully corroborate the views on

* Gazette Medicale de Paris, 12 Janvier, 1850, p. 59.

this subject of Dr. Seymour,* which we noticed at some length in the first number of this Journal. The circumstances under which opium, morphia, belladonna, digitalis, &c., are contra-indicated, must be obvious to every practitioner. The treatment of insanity, however, does not depend solely on the exhibition of medicines; the moral treatment is of as great, and sometimes even of greater, importance. And as we are now exceeding our limits, we shall conclude with the following observations made by Dr. Conolly, in the report before us:—"I consider the direct treatment of any form of insanity by *mere* medical applications, to be very limited; but the indirect treatment of mental maladies by innumerable means acting on the body and the mind, is of immeasurable importance. These means can, I believe, seldom be efficiently applied, except in well-constructed and well-conducted lunatic asylums, superintended by well-educated men, aided by benevolent and active attendants. By such means, I believe many insane persons to be capable of cure; and all, however incurable and hopeless, capable of improvement and relief."†

---

### Art. III.—MORAL STATE OF SOCIETY.‡

* Seymour on Mental Derangement. 2 vols. London, 1847.

† Report, p. 444.

